# The dark side of the moon: can critical health literacy offer solutions to the fundamental problems of health literacy?

**DOI:** 10.1177/17579759241298255

**Published:** 2024-12-06

**Authors:** Susie Sykes, Stephan van den Broucke, Thomas Abel

**Affiliations:** 1 Institute of Health and Social Care, London South Bank University, London, UK; 2 Institut de Recherche en Sciences Psychologiques, Université catholique de Louvain, Louvain-la-Neuve, Belgium; 3 Institute of Social and Preventive Medicine, University of Bern, Bern, Switzerland

**Keywords:** health literacy, critical health literacy, social determinants of health

## Abstract

This commentary is intended as a response to ongoing concerns expressed about fundamental limitations of current research, policy, and practice surrounding health literacy. These concerns emphasise the individualistic and reductionist approaches which often dominate health literacy work, as well as a neglect of broader structural factors in addressing pressing public health issues. The potential of critical health literacy as a concept and practical approach which responds to these critiques is presented. A case is made that critical health literacy, as a concept that operates at both the community and individual level, offers an opportunity to address and eventually overcome these basic limitations in current health literacy approaches.

Health literacy (HL) has gained importance in health care and public health research, policy and practice. While critiques of HL are not new (1), with its increasing popularity, a controversy has grown over its potential to address public health’s biggest questions, most prominently the challenge to reduce health inequities. Critical voices point to the individualistic and reductionist approaches which often dominate HL work and to a neglect of the broader structural factors that are important in addressing pressing public health concerns, such as social inequities or the commercial determinants of health. The Lancet's November 2022 (2) editorial clearly articulated this position, marking a springboard for a much-needed critical discussion.

These concerns have legitimacy, given the focus on functional health literacy that still dominates much of the current HL practices. Functional HL indeed prioritises individual competencies to obtain, process and understand health information to make appropriate health decisions. This focus is somewhat less prominent when HL is understood as a *relational* concept (3) in which those individually held competencies are mediated by the organisational structures and the accessibility of resources within which they operate (4). These ideas are embedded within developing concepts of organisational health literacy (5) and systems health literacy (6). But even if seen as relational, the problems remain when the focus of HL is kept ‘downstream’ on individual action and on functional and communicative aspects. Understanding and acting on the ‘upstream’ determinants of health, which are the root cause of health inequities, is critical for creating lasting and equitable health improvements, reducing health care costs and promoting overall well-being.

Individuals – patients and citizens – also need competencies to deal with challenges that go beyond individual health choices. Managing health in an increasingly complex society implies making critical decisions about, for example: health insurance, patient rights, food choices in profit-driven markets, or linking health decisions to sustainability and climate change. On such issues, as the COVID-19 crisis has shown, approaches are needed that support people’s capacity to understand critically and act on health issues that range from: individual lifestyle changes to community engagement for a healthy environment, and political controversies on the commercial and social determinants of health (7–9). The failure to act collectively to advance climate change, digitalisation or poverty and inequality not only hinders human development but also worsens polarisation and further erodes trust in people and institutions (10).

A concept that has been suggested to overcome these issues of individualisation and neglect of fundamental structural deficits, is critical health literacy (CHL). Building on antecedents of foundational HL skills, health knowledge, advanced cognitive skills, and self-efficacy, CHL focuses on the competencies that are needed to recognise, reflect and act on the social conditions and determinants of health, in a context-specific way (11). It includes but goes beyond the critical appraisal of information (12), as the ‘critical’ element in CHL focuses on a commitment to social justice and political action for change. CHL can operate at both the community and individual levels and incorporates critical reflection on all factors and actors that are involved in the production and distribution of health, including ‘skills in social mobilisation and consumer advocacy’ (4) to take social and political action to challenge them. As such, it directs individual and collective agency explicitly towards social and commercial inequalities in health, while raising critical awareness of social and structural factors that impact health and keeping individual and collective action at its centre.

CHL has been shown to be a useful concept in interventions that act on the social determinants of health (11). It has the potential to align with evolving relational frameworks such as organisational health literacy, where inclusion of CHL encourages active participation in decision-making processes and advocacy for systemic changes that enhance equity and access. Still, there is work to be done to improve our understanding of current levels of CHL across communities and assess the impact of interventions designed to enhance CHL. Developing CHL requires participatory and deliberative strategies based on a commitment to collaboration, dialogue and empowerment and it is clear that these are not without their practical and ideological problems (11). However, as public health is grappling with the challenges of contemporary ‘polycrises’ (13), including persistent and growing inequalities, public health emergencies, and the consequences of climate change, the potential contribution of CHL and agency for health across communities should not be overlooked. Awareness and a basic understanding of the structural drivers of health and the capacity and motivation to act on them are relevant across public health issues. This positions CHL as a crucial mediator of the causes and effects of those determinants (4,14) and as an important part of citizenship for health. Finally, it may help illuminate the parts of the unequal production and distribution of opportunities for health that are currently left in the dark by individualised and functional HL approaches.
